# Insights from Patterns of SARS-CoV-2 Immunoglobulin G Serology Test Results from a National Clinical Laboratory, United States, March–July 2020

**DOI:** 10.1089/pop.2020.0256

**Published:** 2021-02-05

**Authors:** Harvey W. Kaufman, Zhen Chen, William A. Meyer, Jay G. Wohlgemuth

**Affiliations:** Quest Diagnostics, Secaucus, New Jersey, USA.

**Keywords:** COVID-19, SARS-CoV-2

## Abstract

Serologic tests for severe acute respiratory syndrome coronavirus-2 (SARS-CoV-2) provide information on past infection and immune response. To better understand the persistence of immune response and the proportion of the population who can develop one, the authors assessed patterns of immunoglobulin G (IgG) positivity over time in individuals tested for SARS-CoV-2 RNA or IgG at a large national reference laboratory. More than 2.4 million SARS-CoV-2 IgG serology (initiated April 21, 2020) and 6.6 million nucleic acid amplification testing (NAAT) (initiated March 9, 2020) results on persons from across the United States as of July 10, 2020 were analyzed. Additional IgG serology results through August 11, 2020 were used for one household analysis. SARS-CoV-2 IgG positivity was observed in 91% (19,434/21,452) of individuals tested after a positive NAAT result and in 10% (7,831/80,968) after a negative NAAT result. Factors associated with seropositivity include age, region of patient residence, and interval between NAAT and IgG serology. The probability of persistent IgG seropositivity declined from 98.6% after 1 week to 74.3% after 2 months, less so in individuals ages ≥55 years than in younger groups. Specimens within 2 days from pairs of same-household members showed 92% IgG antibody concordance. Household adults were more frequently IgG positive prior to household children testing positive (36% versus 8%). IgG serology testing can identify an immune response to SARS-CoV-2 that varies based on age, sex, and duration since exposure. Loss of detectable IgG seropositivity occurs, in some patients, over weeks or months. Adults may be infecting household children.

## Background

The severe acute respiratory syndrome coronavirus-2 (SARS-CoV-2) illness has spread rapidly as a global pandemic. Widespread, preexisting immunity was presumably lacking in the population upon initial virus infection. The first known case of coronavirus disease 2019 (COVID-19) caused by SARS-CoV-2 was identified in the United States in January 2020,^1^ and a national public health emergency was declared on March 13, 2020. As of late July 2020, the pandemic has resulted in 4.3 million confirmed positive cases and likely more than 150,000 deaths in the United States.^2^

Specific treatments for COVID-19 are being investigated and public health countermeasures, including social distancing, quarantine, and contact tracing, are being implemented. However, controlling the pandemic in the long term likely will depend on sufficient proportions of the population acquiring immunity to the virus, either through natural infection or immunization with an effective vaccine. Studies on identifying appropriate vaccine candidates and vaccine immunogenicity are progressing,^3,4^ but conclusive evidence of acquired immunity and corresponding serological correlates of protection from SARS-CoV-2 reinfection is currently limited. Such correlates require measurement of neutralizing antibodies and virus antigen-specific quantitative immunoglobulin G (IgG) levels. In the interim, the widely available qualitative detection of SARS-CoV-2-specific IgG serves as the primary biomarker of the longer term adaptive immune response.

Evidence from studies with small sample sizes from Asia, Europe, and the United States suggests that most immunocompetent individuals produce serum antibody responses to SARS-CoV-2 infection.^5^ Large-scale clinical and epidemiologic studies, along with studies on convalescent plasma and administration of monoclonal antibodies, are currently underway to assess serological responses following SARS-CoV-2 infection.^6^ In the meantime, useful correlative insights can be gathered from available clinical testing data.

In this study, the research team retrospectively analyzed results from SARS-CoV-2 IgG antibody testing and nucleic acid amplification testing (NAAT) performed at a large national clinical laboratory. The objectives were to (1) estimate the proportion of individuals with positive or negative NAAT results who had evidence of SARS-CoV-2 IgG and identify predictors of SARS-CoV-2 IgG positivity, among the individuals with paired specimens for NAAT and IgG testing; (2) evaluate the probability of persistent IgG seropositivity among those who had an initial positive IgG result, followed by subsequent IgG testing; (3) evaluate IgG antibody concordance of dual household members; and (4) identify the IgG serologic index case (adult or child) among household members.

## Methods

Results from SARS-CoV-2 NAAT and qualitative IgG tests performed at Quest Diagnostics through July 10, 2020, were included in the analysis. The NAAT testing was initiated on March 9 and qualitative IgG antibody testing was initiated on April 21. Results were excluded for patients who were <2 years of age at the time of testing. For the evaluation of within-household adult and child IgG results, the data inclusion period was extended to August 11, 2020. The SARS-CoV-2 NAAT and IgG antibody methods in use at Quest Diagnostics are designated by the US Food and Drug Administration (FDA) for Emergency Use Authorization (EUA). The ribonucleic acid (RNA) testing platforms include (1) Quest Diagnostics laboratory developed test; (2) cobas (Roche Molecular Systems, Inc., Pleasanton, CA); (3) Panther Fusion, (Hologic, Inc., Marlborough, MA); and (4) Aptima (Hologic, Inc.). The IgG antibody testing platforms include (1) Architect (Abbott Laboratories, Inc., Chicago, IL); (2) VITROS (Ortho-Clinical Diagnostics, Inc., Raritan, NJ); and to a limited extent, (3) the EUROIMMUN Anti-SARS-CoV-2 ELISA (IgG) (EUROIMMUN US Inc.). According to the manufacturer's instructions-for-use documents, each of these serology assays have specificities ≥99%. A comparative study of these methods demonstrated >97.5% qualitative concordance across assay platforms.^7^

For paired specimens of NAAT and IgG with IgG collected after NAAT from individuals with multiple testing results over time, the date of the first positive result for each analyte was included in the analysis. When all serial NAAT results were negative, the earliest NAAT date was used for analysis; when all serial IgG tests were negative, the latest IgG test date was used.

To assess persistence of IgG seropositivity, or loss of IgG (ie, “seroreversion”), the research team evaluated results from individuals who had an initial positive IgG result followed by subsequent IgG testing. The first negative IgG result after an initial IgG positive result was defined as an event (seroreversion), and the last positive result (persistent seropositivity) was considered to be a censored observation in Kaplan-Meier product limit estimate.^8^

For IgG serology analyses, household members were identified as being in the same household by having identical residential addresses. For concordance within a pair of individuals in the same household, 2 household members had to be tested within 2 days of each other; for identifying a serologic index case, the household adult (ages 18–64 years) and child (ages 2–17 years) had to have positive results within 30 days of each other, and the positive adult had to be at least 15 years older than the positive child.

The Chronic Condition Indicator (CCI) was used to categorize chronic conditions for individuals with International Classification of Diseases, Tenth Revision codes assigned by the health care provider at the time of a SARS-CoV-2 laboratory order or in the prior 12 months for any test orders. CCI is a tool developed for clinical research as part of the Healthcare Cost and Utilization Project, a Federal-State-Industry partnership sponsored by the Agency for Healthcare Research and Quality.^9^ The 5-state northeast (NE) area (New York, New Jersey, Rhode Island, Connecticut, and Massachusetts) was selected as a unit to represent a geographic area where community transmission of SARS-CoV-2 and confirmed COVID-19 illness was particularly prevalent during the March through May 2020 period.

Chi-square tests were used to assess difference in proportions. Multivariate logistic regression modeling was built via stepwise selection procedure to predict IgG positivity as a function of NAAT status, sex, age, CCI, geographic area of collected specimens, and interval between NAAT and IgG serology testing. Data analyses were performed using SAS Studio 3.6 on SAS 9.4 (SAS Institute Inc., Cary, NC). Kaplan-Meier product limit estimate and survival plot were conducted in R Statistical Software (R Foundation for Statistical Computing, Vienna, Austria).^10^ This Quest Diagnostics Health Trends report was deemed exempt by the Western Institutional Review Board.

## Results

### SARS-CoV-2 NAAT

Results from 6,643,505 SARS-CoV-2 NAATs, performed on 6,006,609 individuals from March 9 to July 10, 2020, were included in the analysis. SARS-CoV-2 NAAT results were derived from individuals who resided in 96.9% (3032/3128) of the United States counties and those counties account for 99.9% of the United States population (2019 United States Census estimate). Among all individuals, 3,344,152 (55.7%) were female; mean (SD) age was 44.6 (19.2) years; 5,366,684 individuals (89.3%) had ≥1 negative NAAT results, 558,565 (9.3%) had ≥1 positive NAAT results, and 81,360 (1.4%) had a combination of positive and negative NAAT results.

### SARS-CoV-2 IgG serology

**FIG. 1.**
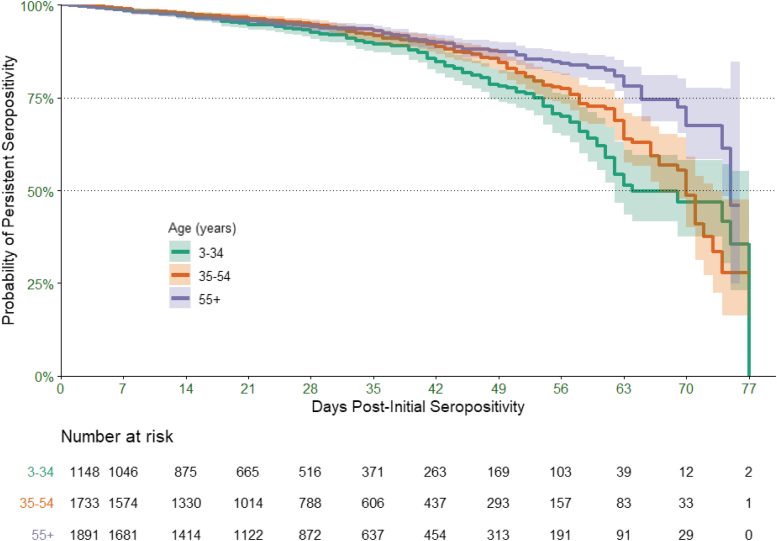
Persistence of seropositivity for SARS-CoV-2 IgG (Kaplan-Meier Curve). Shaded area represents the pointwise 95% confidence interval. IgG, immunoglobulin G; SARS-CoV-2, severe acute respiratory syndrome coronavirus-2.

Results from 2,437,336 SARS-CoV-2 IgG tests, performed on 2,402,282 individuals from April 21 through July 10, 2020, were included in the analysis. Among all individuals, 283,770 (11.8%) had only 1 positive result, 2,083,858 (86.7%) only 1 negative result, 1467 (0.1%) only 1 equivocal result, and 33,187 (1.4%) had ≥2 test results. Among individuals with ≥2 test results, 4772 (51.6% female, mean [SD] age: 49 [16] years) had an initial positive IgG result, followed by subsequent IgG testing. The interval from the first positive result to the first subsequent negative IgG (event) or the last positive result (censored) was 1 to 77 days. Overall, of all individuals with subsequent IgG testing following an initial positive result, 4305 (90.2%) were persistently seropositive and 467 (9.8%) became seronegative over 77 days. The probability of persistent seropositivity declined over time, from 98.6% by the end of the first week to 74.3% by 2 months. Rates of persistent seropositivity did not differ by sex: 75.6% for females versus 72.7% for males by 2 months (*P* = .07). To assess associations of age with persistent seropositivity, individuals were categorized into 3 age groups: 3–34, 35–54, or ≥55 years. Beyond the first month, the probability of persistent seropositivity was significantly higher in patients ages ≥55 years than in younger groups (*P* < .0001) (Figure 1).

### Paired NAAT and IgG serology results

Among 102,420 individuals with paired NAAT/IgG serology specimens with IgG collected after NAAT, 21,452 (20.9%) were positive and 80,968 (79.1%) were negative for SARS-CoV-2 by NAAT. CCIs were available for 14,770 (68.9%) of SARS-CoV-2 NAAT positive and 54,520 (67.3%) of NAAT negative individuals.

**FIG. 2.**
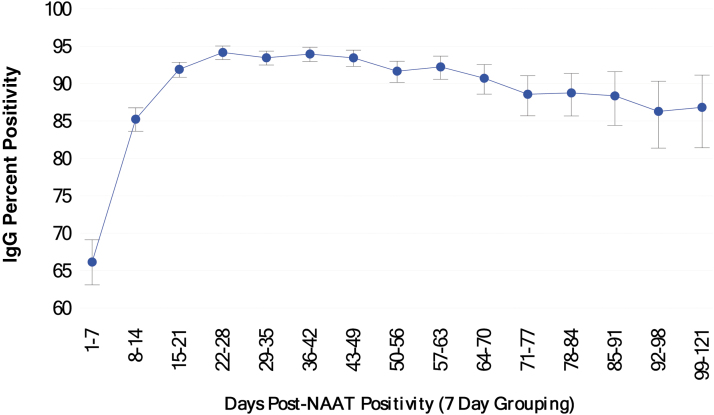
Proportion of individuals testing positive for SARS-CoV-2 IgG, in 7-day groupings, after initial NAAT positivity. Percent positivity equals number positive in the 7-day period/total tested in the 7-day period (bars representing 95% confidence limits). IgG, immunoglobin G; NAAT, nucleic acid amplification testing; SARS-CoV-2, severe acute respiratory syndrome coronavirus-2.

Of individuals with positive NAAT results, 19,434 (90.6%) subsequently had positive IgG results; the mean time to serology testing after NAAT testing was 37.7 days (median 35, range 1–121). As shown in Figure 2, IgG positivity increased from a low of 66.2% 1–7 days after a positive NAAT result and peaked at 22–28 days (94.2% positivity). Although positivity declined gradually over the following weeks, it remained above 86% through 99–121 days. Among the 5577 individuals with an IgG test 15–28 days after their initial positive NAAT result (the period during which humoral immune responses are expected to peak), a total of 5186 (93.0%) demonstrated IgG positivity.

**FIG. 3.**
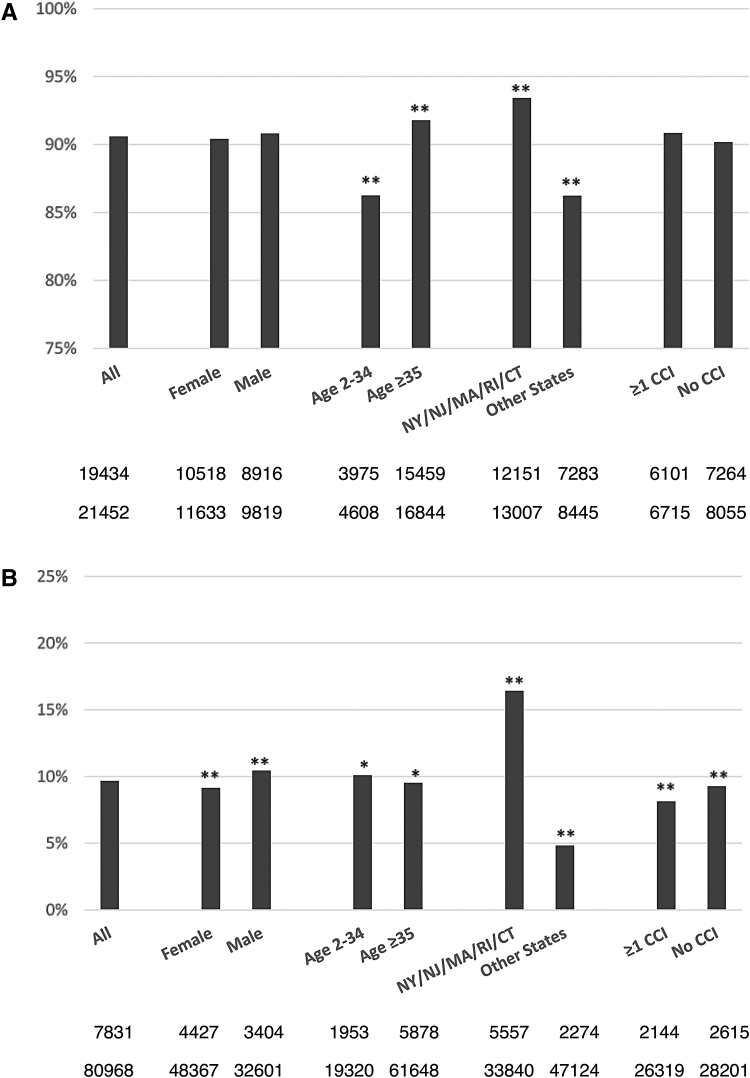
IgG seropositivity rate (percent) among individuals after a SARS-CoV-2 NAAT positive **(A)** or negative **(B)** result. **(A)** First row is number of IgG positives and second row is the total number tested. ** *P* < .0001 from chi-square test for age and state. **(B)** First row is number of IgG positives and second row is the total number tested. ** *P* < .0001 from chi-square test for sex, state, and CCI. * *P* < .02 for age. CCI, chronic condition indicator; IgG, immunoglobin G; NAAT, nucleic acid amplification testing; SARS-CoV-2, severe acute respiratory syndrome coronavirus-2.

The rate of IgG positivity among NAAT-positive individuals was high overall but differed significantly with respect to age (greater in persons ≥35 years of age) and geographic area (higher in the 5-state NE area) (Figure 3A). There were no statistically significant differences by sex or by CCI.

Among all NAAT-negative individuals, the overall rate of subsequent IgG positivity was 9.7% (Figure 3B). In this group, males had a higher IgG positivity rate, as did the younger age group, those from the 5-state NE area, and those without a CCI.

Multivariate logistic regression (Table 1) demonstrated that among SARS-CoV-2 NAAT-positive individuals, IgG positivity was more likely among samples originating from the 5-state NE area and among those from individuals ≥35 years of age. Serology tests performed 22 to 121 days after NAAT positivity had a higher positivity rate than those performed within the first 21 days. In specimens from individuals who were NAAT negative, subsequent IgG positivity was more common in those originating from the 5-state NE area and males (Table 1). A serology test performed ≥22 days after NAAT negativity was less likely to be positive than was a test performed within the first 3 weeks after NAAT negativity. There were marginal differences in IgG positivity by age group but no significant differences between those with or without a CCI, after controlling for other predictors.

**Table 1.** Multivariate Logistic Regression of SARS-CoV-2 Serum IgG Positivity by NAAT Result, with IgG Tested After NAAT

Predictor	Odds ratio estimates (95% confidence limits)	P
**NAAT Positive** (n = 14,770)		
State (NY/NJ/MA/RI/CT vs Other States)	2.0 (1.8, 2.2)	<.0001
Days between NAAT and IgG (22–121 vs 1–21)	2.0 (1.7, 2.2)	<.0001
Age in years (≥35 vs 2–34)	1.8 (1.6, 2.1)	<.0001
Sex (M vs F)	1.1 (1.0, 1.2)	.2
CCI^*^ (≥1 vs None)	1.0 (0.9, 1.1)	.8
**NAAT Negative** (n = 54,517)		
State (NY/NJ/MA/RI/CT vs Other States)	3.6 (3.4, 3.9)	<.0001
Days between NAAT and IgG (22–121 vs 1–21)	0.8 (0.8, 0.9)	<.0001
Age in years (≥35 vs 2–34)	1.1 (1.0, 1.2)	.08
Sex (M vs F)	1.1 (1.1, 1.2)	.0004
CCI^*^ (≥1 vs None)	1.0 (0.9, 1.0)	.2

Logistic regression modeling IgG positive vs. IgG negative with all predictors in the model, stratified by NAAT result.

^*^ Compare ≥1 CCI vs. no CCI.

CCI, chronic condition indicator; IgG, immunoglobulin G; NAAT, nucleic acid amplification testing; SARS-CoV-2, severe acute respiratory syndrome coronavirus-2.

Separately, of a total of 349,528 individuals who had NAAT and IgG specimens collected on the same day, 8434 (2.4%) had positive NAAT results. Of these, 5619 (66.6%) were simultaneously positive for IgG. Of the 341,094 NAAT-negative individuals, 55,170 (16.2%) were simultaneously positive for IgG. In a multivariate logistic model (Table 2), among SARS-CoV-2 NAAT-positive individuals, a simultaneous positive IgG result was more likely in individuals who were ≥35 years of age, were from the 5 NE states, or had ≥1 CCI. Among SARS-CoV-2 NAAT-negative individuals, a simultaneously positive IgG result was more likely in those who were ≥35 years of age, were from the 5 NE states, had ≥1 CCI, or were male.

**Table 2.** Multivariate Logistic Regression of SARS-CoV-2 Serum IgG Positivity by NAAT Result, for Simultaneous IgG and NAAT Testing

Predictor	Odds ratio estimates**(95% confidence limits)	P
**NAAT Positive** (n = 3868)		
State (NY/NJ/MA/RI/CT vs Other States)	10.3 (8.8, 12.0)	<.0001
Age in years (≥35 vs 2–34)	1.7 (1.4, 2.0)	<.0001
Sex (M vs F)	1.1 (0.9, 1.3)	.4
CCI^*^ (≥1 vs None)	1.5 (1.3, 1.8)	<.0001
**NAAT Negative** (n = 186,301)		
State (NY/NJ/MA/RI/CT vs Other States)	6.8 (6.4, 7.2)	<.0001
Age in years (≥35 vs 2–34)	1.1 (1.1, 1.2)	<.0001
Sex (M vs F)	1.0 (1.0, 1.1)	.007
CCI^*^ (≥1 vs None)	1.2 (1.2, 1.3)	<.0001

Logistic regression modeling IgG positive versus IgG negative with all predictors in the model, stratified by NAAT result.

^*^ Compare ≥1 CCI vs. no CCI.

CCI, chronic condition indicator; IgG, immunoglobulin G; NAAT, nucleic acid amplification testing; SARS-CoV-2, severe acute respiratory syndrome coronavirus-2.

### Household IgG serology testing

Among the 134,791 pairs of individuals with the same address who were tested for SARS-CoV-2 IgG within 2 days of each other, 113,718 (84.4%) pairs were both negative, 10,314 (7.7%) were both positive, and 10,759 (8.0%) were discordant.

Among the 4021 households with ≥1 IgG-positive child and ≥1 IgG-positive adult tested within 30 days of each other, the first adult and child tested positive at the same time in 2249 (55.9%) households; the adult had the first positive result in 1435 (35.7%); and the child had the first positive result in 337 (8.4%).

## Discussion

The results of this study demonstrate a high rate of IgG seropositivity in individuals with previously detected SARS-CoV-2 RNA. However, a sizable portion of IgG positive patients can lose detectable IgG seropositivity over a period of weeks or months. In households with both an adult and child testing positive for IgG at different times, the adult tested positive first more often than the child.

Based on analysis of testing volume distribution by counties, the geographic reach of Quest Diagnostics NAAT testing covers virtually the entire US population. The gap in counties not represented by testing (3%) with 0.1% of the United States population may represent those that had a lower test ordering frequency or had testing performed by other clinical laboratories. Alternatively, a low number of test requests also could represent infrequently suspected COVID-19 cases.

SARS-CoV-2 NAAT testing is effective in identifying SARS-CoV-2 RNA from intact virus particles or, in many cases, nucleic acid remnant materials.^11^ Typically, SARS-CoV-2 NAAT is positive a few days after infection initiation and persists for up to 2 weeks or sometimes longer.^11–13^ A negative NAAT result from individuals who are actually infected could be caused by inadequate or improper specimen collection, specimen instability prior to testing, or presence of viral RNA below the level of detection by an assay platform.

IgG antibody is usually detectable starting 1 to 3 weeks after symptom onset.^11^ The presence of IgG positivity may suggest decreased viral infectiousness^14^ and may provide immune protection for an undetermined period of time.^15^ Depending on the timing of SARS-CoV-2 NAAT and IgG serology specimen collection, results can be simultaneously positive. This study shows that the number of simultaneous positive paired NAAT/IgG (349,528) is more than 3 times that of the paired NAAT/IgG with IgG collected after NAAT (102,420). Given that many COVID-19 patients are asymptomatic or mildly symptomatic and the precise time of exposure to an infectious individual is unknown, the dual ordering of NAAT and IgG serology suggests that many health care providers are simultaneously investigating the possibility of both past and present infection.

When the timing of a serum specimen collection is appropriate, a negative SARS-CoV-2 IgG serology result is consistent with absence of prior infection or seroreversion and positive IgG serology is consistent with past infection or recent exposure when the NAAT result is also positive. This role expands the guidelines of the Infectious Disease Society of America, which support “evaluation of patients with a high clinical suspicion for COVID-19 when molecular diagnostic testing is negative and at least two weeks have passed since symptom onset,” because, often, the time of exposure or symptoms is unknown.^16^

Factors associated with seropositivity consistently include age and residency in the NE area versus elsewhere in the United States. Sex and interval between NAAT and IgG serology are observed to be predictors when data are available and sample size is large. These observations regarding age and sex are consistent with those from other studies.^11^ CCI may relate to severity of disease among those infected. However, in this study, CCI was not associated with the likelihood of seropositivity in sequentially collected NAAT and IgG specimens, after adjusting for the interval between NAAT and IgG serology and other potential predictors.

Present study observations may corroborate the findings of Ibarrondo and colleagues,^17^ who reported rapid decay of SARS-CoV-2 antibodies in patients with mostly mild illness. However, loss of detectable IgG seropositivity does not always mean loss of seroprotection to reinfection. For example, IgG antibodies near the assay detection level could represent false-negative results and/or biological variations in antibody levels. Further, patients who have lost detectable IgG may have a robust secondary immune response when reexposed because of immune system memory and cellular responses, which requires further evaluation.^18,19^ Nevertheless, antibody persistence often serves as an indication of protection.^20^

Present study observations suggest that younger people, who are more likely to have mild disease, are losing detectable IgG antibody faster than older individuals. A possible explanation is that older people are more likely to have been exposed to other coronaviruses and SARS-CoV-2 triggers a strong amnestic immune response^19^ or to have more severe COVID-19 disease. Long-term clinical and laboratory evaluations are needed to better define the immune response necessary for COVID-19 immune protection for patients of all ages.

To the research team's knowledge, this is the first large-scale report on SARS-CoV-2 IgG serology testing within households. A priori, the likelihood of demonstrating seropositivity for each household member should be the same 90.6% that was observed overall, if the risk of seropositivity was independent for each household member. This suggests that the statistical probability of IgG antibody positive concordance (positive/positive) is 82.1% (90.6% x 90.6%), the probability of discordance (positive/negative or negative/positive) is 17.0% (2 x 90.6% x 9.4%), and the probability of negative concordance (after both with presumed infection) is 0.9% (9.4% x 9.4%). Thus, the observed household discordance rate of 8.0% is only half of what one would have expected on a statistical basis (8.0% versus 17.0%). This observation may have a similar underlying cause as was noted for the 5 NE states with higher rates of SARS-CoV-2 IgG seropositivity compared to the other states. High viral load and duration of exposure may account for this difference in the NE area and also may apply to the higher-than-expectation discordance rate within households.

Finally, the research team evaluated both adult and child household members with IgG positivity within each age cohort. In most cases, both members of the pair tested positive at the same time. However, among pairs in which one member tested positive before the other, the adult was much more likely to test positive first. This suggests that adults are more likely to be infected prior to children in the same household, which is not surprising, as adults are more likely to work outside the home. However, it may be that children with milder disease than adults are less likely to demonstrate detectable seropositivity, or that children are more likely to display seroreversion if tested earlier.^20–22^ Alternatively, adults may be more likely to be a person of concern based on an exposure or new symptoms. The role of contact tracing to identify secondary household members also must be considered. Of note, the findings of this study reflect a time frame when children were home from school because of school closures or summer recess; the analysis did not include the start of the academic year, when some school systems reopened with full-time classrooms or in a hybrid model.

### Limitations

As an observational study of laboratory test orders and results, the main limitation is that the clinical course of illness and epidemiological history of exposure in the individuals tested were not available. Also, there is no knowledge of whether the testing (NAAT and serology) was performed for clinical care, screening, or public health surveillance activities. The primary strength of this investigation is the enormous scale of data from across the United States and the ability to link results longitudinally for individuals and within households.

## Conclusions

In the largest study to date of paired molecular diagnostic and subsequent serology tests for SARS-CoV-2, the research team found that 90.6% of individuals with NAAT-positivity subsequently had evidence of IgG antibody by qualitative testing using a laboratory-based FDA EUA platform. Of interest, 9.7% of those who tested negative for SARS-CoV-2 RNA NAAT subsequently tested positive for IgG. The results of simultaneous NAAT and IgG serology testing revealed IgG positivity in 66.6% of patients with positive NAAT results and in 16.2% of those with negative NAAT results. This observed IgG positivity is hopefully of clinical value, in that these patients likely represent diagnostic challenges to their physicians.

The combined results of both tests can reveal if there is current infection or past exposure with an immune response. People are exposed at different viral loads and durations, and the immune response varies. Further, specimen collection events occur at different times relative to exposure and development of symptoms. The relatively high frequency of infection based only on a positive SARS-CoV-2 IgG antibody result creates challenges to understanding the dynamics affecting NAAT and IgG serology result interpretation. This uncertainty reinforces the position that maximal diagnostic utility is achieved when both a NAAT and a simultaneous or subsequent IgG serology test are performed on at-risk patients when exposure history is unknown. Notwithstanding the value of dual testing, caution is warranted in test interpretation given the variability in the human immune response, the limitations of testing highlighted in this study, and our collective expanding understanding of SARS-CoV-2.

Prevalence of SARS-CoV-2 in the community in the NE states likely contributed to the higher seropositivity rate among NAAT-negative individuals in this region. Negative NAAT results in association with a positive IgG result may be related to timing and other factors. While interpretation of SARS-CoV-2 seropositivity is being addressed, this study provides support for SARS-CoV-2 antibody testing as a marker of subclinical or medically-attended infection, in both high- and low-prevalence areas of reported COVID-19 illness. Public health surveillance must incorporate the underlying expected seropositivity and seroreversion rates while evaluating community rates of infection.

The presence of SARS-CoV-2 IgG in serum has not yet been confirmed to be immunologically protective against reinfection in humans. Studies are needed to address critical questions regarding the correlates of immunity to SARS-CoV-2 in those who are asymptomatically infected as well as those who have experienced symptomatic infection, individuals of all ages, vulnerable populations, and in special populations such as first responders, health care workers, pregnant women, and the Black non-Hispanic and Latinx populations.
